# Accumulation of phenanthrene by roots of intact wheat (*Triticum acstivnm *L.) seedlings: passive or active uptake?

**DOI:** 10.1186/1471-2229-10-52

**Published:** 2010-03-22

**Authors:** Xin-Hua Zhan, Heng-Liang Ma, Li-Xiang Zhou, Jian-Ru Liang, Ting-Hui Jiang, Guo-Hua Xu

**Affiliations:** 1 College of Resources and Environmental Sciences, Nanjing Agricultural University, Nanjing, Jiangsu Province, 210095, PR China; 2 Current address: Greenstar Plant Products Inc, 9430 198 St, Langley, BC, V1M 3C8, Canada

## Abstract

**Background:**

Polycyclic aromatic hydrocarbons (PAHs) are of particular concern due to their hydrophobic, recalcitrant, persistent, potentially carcinogenic, mutagenic and toxic properties, and their ubiquitous occurrence in the environment. Most of the PAHs in the environment are present in surface soil. Plants grown in PAH-contaminated soils or water can become contaminated with PAHs because of their uptake. Therefore, they may threaten human and animal health. However, the mechanism for PAHs uptake by crop roots is little understood. It is important to understand exactly how PAHs are transported into the plant root system and into the human food chain, since it is beneficial in governing crop contamination by PAHs, remedying soils or waters polluted by PAHs with plants, and modeling potential uptake for risk assessment.

**Results:**

The possibility that plant roots may take up phenanthrene (PHE), a representative of PAHs, via active process was investigated using intact wheat (*Triticum acstivnm L*.) seedlings in a series of hydroponic experiments. The time course for PHE uptake into wheat roots grown in Hoagland solution containing 5.62 μM PHE for 36 h could be separated into two periods: a fast uptake process during the initial 2 h and a slow uptake component thereafter. Concentration-dependent PHE uptake was characterized by a smooth, saturable curve with an apparent *K*_m _of 23.7 μM and a *V*_max _of 208 nmol g^-1 ^fresh weight h^-1^, suggesting a carrier-mediated uptake system. Competition between PHE and naphthalene for their uptake by the roots further supported the carrier-mediated uptake system. Low temperature and 2,4-dinitrophenol (DNP) could inhibit PHE uptake equally, indicating that metabolism plays a role in PHE uptake. The inhibitions by low temperature and DNP were strengthened with increasing concentration of PHE in external solution within PHE water solubility (7.3 μM). The contribution of active uptake to total absorption was almost 40% within PHE water solubility. PHE uptake by wheat roots caused an increase in external solution pH, implying that wheat roots take up PHE via a PHE/nH^+ ^symport system.

**Conclusion:**

It is concluded that an active, carrier-mediated and energy-consuming influx process is involved in the uptake of PHE by plant roots.

## Background

Polycyclic aromatic hydrocarbons (PAHs) are a group of organic compounds composed of two or more fused aromatic rings in linear, angular, or cluster arrangements [1]. PAHs are of particular concern because of their hydrophobic, recalcitrant, persistent, potentially carcinogenic, mutagenic and toxic properties, and their ubiquitous occurrence in the environment.

Over 90% of PAHs in the environment reside in surface soil [2]. Furthermore, plants grown in PAH-contaminated soils can become contaminated with PAHs due to their absorption [3-6]. Therefore, they may pose human and animal health hazards. Previous studies have shown that dietary intake of PAHs can be a significant route of exposure to the general population where vegetables and grains are a major source of dietary PAHs [7,8]. It is thus important to understand exactly how PAHs are transported into the plant root system and into the human food chain, since it is helpful to produce PAH-free crop products by means of genetic engineering, to remove PAHs from PAH-polluted soils or water through phytoremediation, and to model potential uptake for risk assessment.

Over the last three decades, organic compounds uptake by roots, particularly pesticides/herbicides, has been widely studied [9-14]. Plant roots can take up organic contaminants via passive diffusive partitioning (i.e. apoplastic) and/or active (i.e. symplastic) process, depending on the properties of the organic contaminant and the plant species. Passive transport proceeds in the direction of decreasing chemical potential; nevertheless, active transport is against the chemical potential gradient, requiring expenditure of energy [15]. The absorption of non-ionized organic compounds by roots of higher plants is generally thought to be a passive, diffusive, partitioning and nonmetabolic process [16]. Uptake of non-ionized organic compounds is influenced by the properties of the contaminant [17,18]. Briggs *et al*. [9] established a linear relationship between the octanol/water partition coefficient (*K*_OW_) of non-ionized chemicals and the observed root concentration factor (RCF, i.e., chemical concentration in the root/concentration in external solution) from their experiments involving in the uptake of Omethylcarbamoyloximes and substituted phenylureas into barley plants. With consideration of the passive transport of non-ionic organic pollutants into plants (including crops), a partition-limited model has been proposed to estimate the concentration of a contaminant in plants [19]. However, during further test of the partition-limited model through uptake of PAHs by plant roots, some researchers observed that predicted and measured values of PAH content in plant roots fitted well at low PAH concentrations in soils or hydroponic solution, whereas the prediction error was considerably large, with a maximum of more than 81%, at high PAH concentration in soils or hydroponic solution [20,21]. Collins *et al*. [22] pointed out that looking at root uptake of non-ionic organic chemicals purely as a partitioning process may be incorrect as this assumes it is independent of concentration, which it is not when the roots become saturated. Wild *et al*. [23] visualized the occurrence of anthracene and phenanthrene within the root cell vacuole using two-photon excitation microscopy. All of the above-mentioned seem to indicate that the passive uptake cannot satisfactorily account for the transport process of PAHs into plant roots.

Despite much work regarding uptake and accumulation of non-ionized organic compounds like PAHs in plants conducted previously, mechanisms for PAHs uptake by plant roots and translocation in plants still remain unclear, in particular, whether active transport is involved in root uptake of PAHs and what the proportion of active transport to the total uptake of PAHs by roots is.

In this paper, it is hypothesized that there are two general mechanisms for PAHs uptake and transport, i.e. a passive and an active component coexisting in higher plants with their relative contribution being dependent much upon the plant species and PAH levels. The objectives of this study are i) to confirm whether active transport is involved in the uptake of PAHs by plant roots and ii) to evaluate the relative contribution of active and passive components with respect to PAHs uptake and transport processes in higher plants. To our knowledge, this is the first report to demonstrate that an active component is involved in PAHs uptake.

## Results

### Absorption of PAHs as a function of time

**Figure 1:**
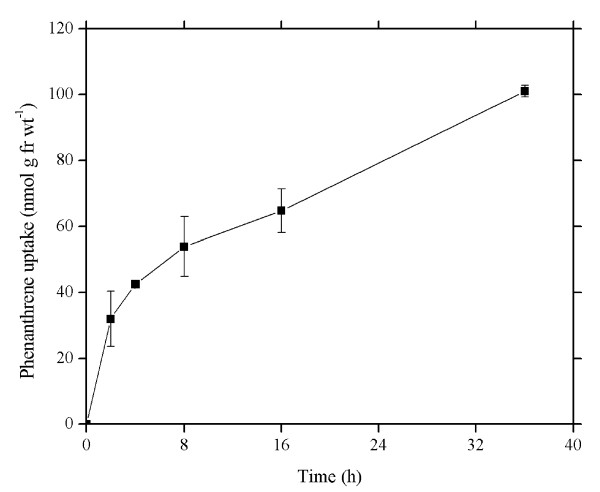
**Time course of phenanthrene uptake by roots of wheat seedlings through 36 h**. Roots were incubated in Hoagland nutrient solution (pH 5.5) containing 5.62 μM phenanthrene at 25°C. Data points represent mean and SD values of triplicates. Error bars do not extend outside all data points. fr wt, Fresh weight.

To investigate uptake of PHE, wheat roots were exposed to 5.62 μM PHE for differing lengths of time and the amount of PHE that passed into the root was determined. Figure 1 shows the time-dependent PHE accumulation in roots of intact wheat seedlings. PHE uptake by wheat roots was nonlinear over 36 h. Wheat roots continued to accumulate PHE through 36 h. A characteristic immediate, rapid rate of uptake was followed by continued uptake but at a decreasing rate. PHE uptake rates between 0 (the initial) and 2 h, and 2 and 36 h were 16.0 ± 4.18 and 2.03 ± 0.06 nmol g^-1 ^fr wt h^-1^, respectively. PHE uptake rate in the first period (0 to 2 h) was almost 8 times higher than that in the period of 2 to 36 h.

### Effect of temperature

**Figure 2:**
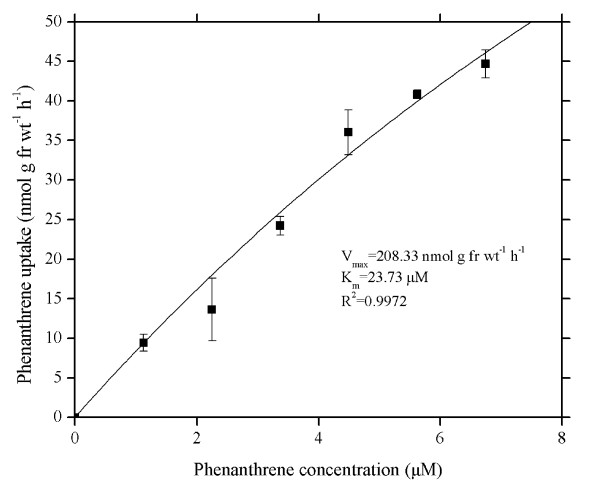
**Concentration dependence of phenanthrene uptake into intact wheat roots**. Phenanthrene concentrations varied from 0 to 6.74 μM in Hoagland nutrient solutions (pH 5.5). Data points represent mean and SD values of triplicates. Error bars do not extend outside all data points. fr wt, Fresh weight.

Concentration-dependent uptake of PHE by wheat roots has features of saturating kinetics within the range of 7.3 μmol L^-1^, PHE water solubility (Figure 2). The curve can be described with Michaelis-Menten equation:(1)

Where *V*_max _(nmol g^-1 ^fresh weight h^-1^) is the maximal transport rate when all available carrier sites are loaded, *C *(μM) is the external concentration of phenanthrene, *K*_m _(μM) is the Michaelis constant, equal to the substrate concentration giving half the maximal transport rate. Generally, the lower the *K*_m _value, the stronger the affinity between carrier and substrate carried [15]. The apparent *K*_m _value (derived from Lineweaver-Burk data transformations) for the saturable curve was 23.7 μM, significantly higher than the PHE solubility in water (7.3 μM, at 25°C). The *V*_max _value was 208 nmol g^-1 ^fresh weight h^-1^.

**Figure 3:**
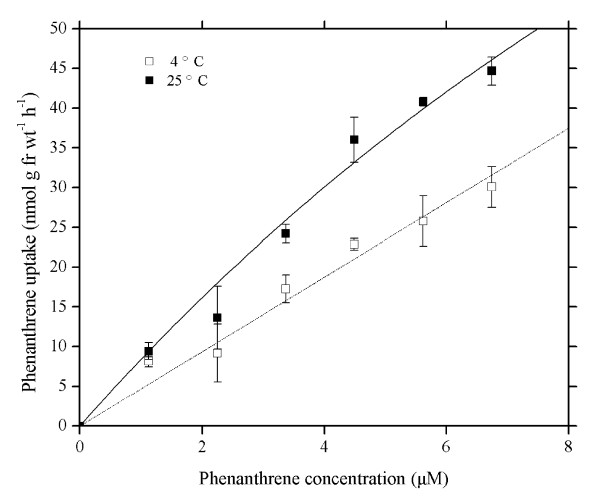
**Concentration-dependent uptake of phenanthrene at 25 and 4°C in intact wheat roots**. Phenanthrene concentrations in Hoagland nutrient solutions (pH 5.5) ranged from 0 to 6.74 μM. Data points represent mean and SD values of triplicates. Error bars do not extend outside all data points. fr wt, Fresh weight.

The uptake of PHE was significantly temperature-dependent (Figure 3). Absorptions of PHE at 25°C were markedly higher than at 4°C (paired *t*-test, 95% confidence level), suggesting that low temperature (4°C) inhibited the uptake of PHE by wheat roots compared with PHE uptake at 25°C. The inhibition by low temperature became stronger with an increase in PHE concentration in hydroponic solution. The inhibition effect of the low temperature was slight for the treatment with PHE concentrations of 0-2.25 μM, whereas the inhibition effect was strengthened for the treatment with PHE concentrations of 2.25-6.74 μM. The inhibition rate, i.e., (uptake of PHE at 25°C - uptake of PHE at 4°C) × 100/uptake of PHE at 25°C, ranged from 13.70% to 36.87%.

### Effect of 2,4-dinitrophenol

**Figure 4:**
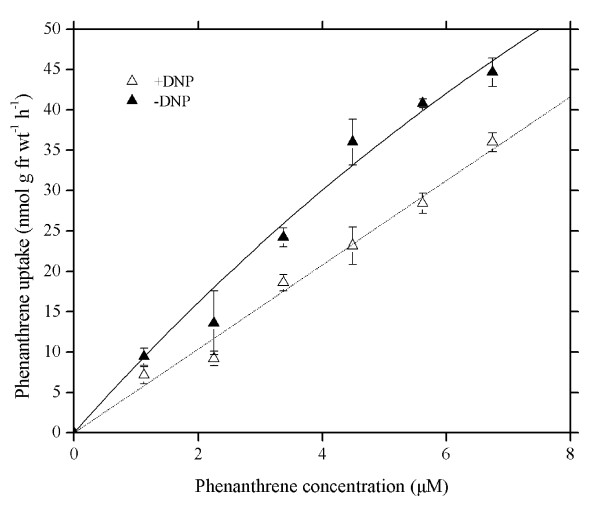
**Concentration-dependent uptake of phenanthrene in intact wheat roots with the presence and absence of 2,4-dinitrophenol (1 mM), a common metabolic inhibitor**. Phenanthrene concentrations in Hoagland nutrient solutions (pH 5.5) ranged between 0 and 6.74 μM. Data points represent mean and SD values of triplicates. Error bars do not extend outside all data points. fr wt, Fresh weight. DNP, 2,4-dinitrophenol.

In general, dinitrophenol (DNP) is considered primarily as an uncoupler of oxidative (and, to a lesser extent, photosynthetic) phosphorylation, and of proton-coupled fluxes at the plasma membrane and endomembranes via the dissipation of transmembrane electrochemical gradients of protons. The effect of 2,4-DNP, a common metabolic inhibitor, upon the uptake of PHE was investigated in this study. Uptake of PHE was strongly depressed by 1 mM 2,4-DNP but was not completely inhibited (Figure 4). The decreased PHE uptake induced by 2,4-DNP was statistically pronounced with paired *t*-test, at 95% confidence level. Similar to the inhibition effect of low temperature on uptake of PHE, the depression effect of 2,4-DNP also exhibited a dependence on PHE concentration. At PHE concentrations of 0-2.25 μM, the inhibition effect of 2,4-DNP was lighter. But the inhibition effect of 2,4-DNP was much stronger at PHE concentrations of 2.25-6.74 μM with rates between 19.47% and 35.60% (Figure 4).

### Effect of naphthalene

**Figure 5:**
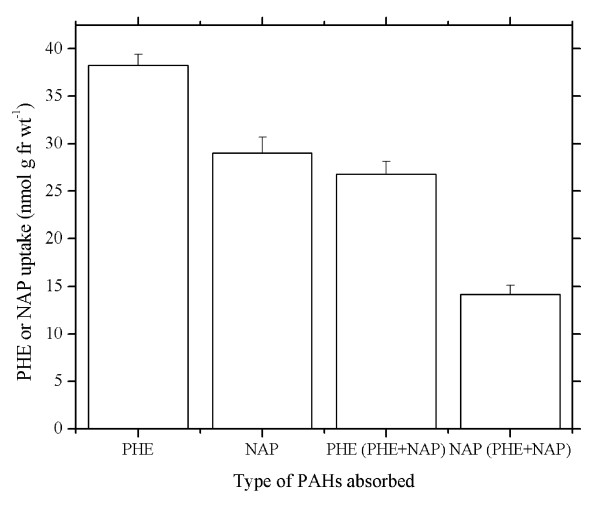
**Uptake competition between phenanthrene and naphthalene in wheat roots**. Hydroponic solution was Hoagland nutrient solution (pH 5.5). PHE, Hoagland solution contained 2.81 μM phenanthrene. NAP, Hoagland solution contained 3.91 μM naphthalene. PHE (PHE+NAP), Hoagland solution contained 2.81 μM phenanthrene and 3.91 μM naphthalene, and phenanthrene uptake was detected. NAP (PHE+NAP), Hoagland solution contained 2.81 μM phenanthrene and 3.91 μM naphthalene, and naphthalene uptake was detected. Data points represent mean and SD values of triplicates. fr wt, Fresh weight. PAHs, Polycyclic aromatic hydrocarbons. PHE, Phenanthrene. NAP, Naphthalene.

Most of the studies regarding PAH uptake by plant roots have hitherto focused on an individual PAH [23-25]. However, PAH uptake by plant roots with the presence of two or more types of PAHs is less addressed. PHE uptakes by wheat roots with the absence and presence of naphthalene (NAP) are presented in Figure 5. Although the water solubility of NAP (247.3 μmol L^-1^) is much higher than that of PHE (7.3 μmol L^-1^) [26], wheat roots took up more PHE than NAP (Figure 5) with PHE uptake 1.32 times more than that of NAP. The presence of NAP inhibited PHE uptake, in turn, PHE also inhibited NAP uptake. Uptake of PHE decreased by as much as 70.1% with the presence of NAP, whereas NAP uptake decreased by 51.2% in the presence of PHE. The inhibition rate exhibited a similar trend. The inhibition rate of PHE uptake by NAP, i.e., (uptake of PHE by wheat roots treated with PHE - uptake of PHE by wheat roots treated with PHE and NAP) × 100/uptake of PHE by wheat roots treated with PHE, was 29.9%, and that of NAP uptake by PHE, i.e., (uptake of NAP by wheat roots treated with NAP - uptake of NAP by wheat roots treated with PHE and NAP) × 100/uptake of NAP by wheat roots treated with NAP, was 48.8%. Both decrease in uptake and the inhibition rate revealed that the inhibition of NAP uptake by PHE was stronger than that of PHE uptake by NAP. Therefore, competitive inhibition occurs when 2 or more types of PAH are present in culture solution.

### Effect of pH

The interaction between protons (H^+^) and other cations or anions is of general importance for plant mineral nutrition. Thus, external solution pH has received much attention during absorption of compounds by plant roots [27,28]. In the present work, PHE uptake by wheat roots caused significant increase in external nutrient solution pH (Table 1). Moreover, ΔpH (i.e., nutrient solution pH after 4 h of PHE uptake minus the initial nutrient solution pH) increased with the increase of PHE uptake by wheat roots. For example, ΔpH is 0.21 in the control, about a 2-fold increase in the treatment with 2.81 μM PHE, and an increase factor of 3 in the treatment with 5.62 μM PHE. In the control, the increase in nutrient solution pH resulted from a NO_3_^-^/H^+ ^symport [29]. Obviously, nutrient solution pH increase in the other two treatments was caused by PHE uptake when compared with the control. The change in pH during uptake of phenanthrene by wheat roots in Millipore water further confirmed this phenomenon (Table 1).

**Table 1:** pH values of hydroponic solution initially and after 4 h of PHE uptake

Treatment	Hoagland nutrient solution			Millipore water		
	
	**pH**_**1**_	**pH**_**2**_	ΔpH	**pH**_**1**_	**pH**_**2**_	ΔpH
+0 μM PHE	5.50	5.71 ± 0.02 c	0.21 ± 0.02 c	5.50	5.47 ± 0.04c	-0.03 ± 0.04c
+2.81 μM PHE	5.50	5.90 ± 0.03 b	0.40 ± 0.03 b	5.50	6.08 ± 0.03b	0.58 ± 0.03b
+5.62 μM PHE	5.50	6.15 ± 0.04 a	0.65 ± 0.04 a	5.50	6.24 ± 0.05a	0.74 ± 0.05a

Hoagland nutrient solution (pH 5.5) and Millipore water (pH 5.5) were employed for hydroponic solution. a, b, c, *p *< 0.05. PHE, phenanthrene. pH_1_, pH_initial_. pH_2_, pH_after 4 h_. ΔpH = pH_after 4 h _- pH_initial_.

## Discussion

In contrast to uptake of ions by plant roots, mechanisms for uptake of non-ionic organic chemicals like PAHs still remain poorly understood. Chemicals may enter plant roots through passive and/or active process and this process is much dependent on the types of plant and chemical, and chemical level in solution. Passive uptake is a nonmetabolic, 'downhill' process, driven by diffusion or mass flow. However, active uptake is an 'uphill', energy-consuming process against the gradient of potential energy. It is generally accepted that the uptake of anthropogenic organic chemicals by plant roots is a passive, diffusive process [16,22,30]. Thus, the uptake of PAHs by plant roots is assumed to be a passive, partitioning process [31]. Actually, the process of PAHs absorption by plant roots is considerably complex and little information about mechanisms for PAHs uptake by the plant roots addressed is available.

Kvesitadze *et al*. [32] have reported that roots absorb environmental contaminants in two phases: in the first fast phase, substances diffuse from the surrounding medium into the root; in the second they gradually distribute and accumulate in the tissues. In this study, the time course of PHE uptake within 36 h can be divided into two parts (Figure 1): a fast influx period (the initial to 2 h) and a slow influx period (2 to 36 h). During the fast period, sorption by root cell wall, diffusion and transpiration flow cause a high rate of PHE uptake. Gao *et al*. [33] have also found an initial rapid uptake phase of PHE by ryegrass. Nevertheless, the low rate of PHE uptake after the initial rapid uptake phase is mainly attributable to passage into root cytoplasm and vacuole. The time course of PHE uptake by wheat roots is consistent with that of glyphosate uptake by suspension-cultured potato [34]. Denis and Delrot [35], and Tilquin *et al*. [36] have observed that glyphosate uptake by broad bean and *Catharanthus roseus *cells is an active process. This seems to imply that the active process is involved in PHE uptake by wheat roots.

To further understand the uptake process for PHE in wheat roots, the investigations with respect to the effects of temperature and inhibitor were conducted. PHE uptake by plant roots is related to its concentration [37]. In the concentration range of PHE studied, PHE uptake is best explained by a single saturable system with a *K*_m _of 23.7 μM and a *V*_max _of 208 nmol g^-1 ^fresh weight h^-1^(Figure 2). Denis and Delrot [35] have found that phosphate uptake by broad bean is characterized by a saturating nature. Hart *et al*. [38] have also observed the similar characteristic in paraquat uptake by roots of intact maize seedlings. They attributed the saturable kinetics to uptake via a carrier-mediated process due to the kinetics of ion or molecule transport through membranes of plant cells like the relationship between an enzyme and its substrate, using terms of enzymology. Our result is in agreement with those reported by Denis and Delrot [35], and Hart *et al*. [38], which indicates that the carrier-mediated process exists during PHE uptake by wheat roots. Naphthalene is a PAH with two benzene rings, and phenanthrene consists of three condensed benzene rings. The competitive effect of PHE and NAP upon root uptake may appear on account of their similar physico-chemical properties (Figure 5). Reciprocal inhibition in root uptake between PHE and NAP further suggests that PAH uptake by wheat roots proceeds with a carrier-mediated system, and NAP and PHE share a common transport mechanism.

The fact that uptake is not affected by temperature is an indicator that the compounds are retained by physical sorption rather than biochemically [16] and metabolically coupled membrane transport processes may be inhibited by low temperature [39], whereas physical processes such as adsorption and diffusion are only slightly affected by temperature [15,39]. Moreover, Hart *et al*. [40] have pointed out that the difference in ion levels measured in intact roots at 23°C and in intact roots incubated at low temperature (2°C) can represent ion taken up across the root plasma membrane. The results presented in Figure 3 display that the rate of PHE absorption at 4°C was a part of the rate at 25°C. The reduction in absorption of PHE by low temperature increased with increasing external PHE concentration in hydroponic solution. A similar effect of low temperature upon Zn^2+ ^uptake was found previously for sugarcane leaves [27], barley roots [41] and wheat roots [40,42]. In addition, Liang *et al*. [43] also found Si uptake by cucumber roots was inhibited by low temperature. The above authors interpreted these findings as evidence of the metabolic control of absorption. In the present study, we found that lower temperature of 4°C didn't result in a distinct decrease of PHE apparent solubility in Hoagland nutrient solution containing 0.1% methanol as compared to the temperature at 25°C. Therefore, the reduction in phenanthrene uptake by wheat root at low temperature is not due to the decrease in phenanthrene apparent solubility caused by low temperature. Figure 4 shows that 2,4-DNP may inhibit PHE uptake by wheat roots and the inhibition effect of 2,4-DNP on PHE uptake is gradually strengthened with increasing external PHE concentration in the culture solution. The inhibitions by 2,4-DNP and by low temperature are approximately even, with inhibition rate up to almost 40%. The data from Figure 3 and 4 suggest the existence of metabolic mediation in the PHE absorption process.

PHE uptake by wheat roots results in an increase in external solution pH (Table 1). Furthermore, the larger the amount of PHE taken up by wheat roots, the higher external solution pH. However, the passive absorption process cannot satisfactorily explain the phenomenon that occurs during the absorption of PHE (a non-ionic and hydrophobic organic compound). It is well known that the NO_3_^-^/H^+ ^symport can cause an increase in external solution pH [15]. Williams *et al*. [44] and Noiraud *et al*. [45] have reported that sucrose transport across the plasma membrane is a sucrose-H^+ ^symport process. Similar to sucrose, PHE is present in solution as a form of molecule. We presume that PHE influx may be coupled with H^+ ^influx and that PHE transport across the plasma membrane proceeds via a PHE/nH^+ ^symport mechanism. Although the hypothesis of PHE/nH^+ ^symport remains to be further tested, the change in external solution pH during root uptake of PHE indicates that active absorption is involved in PHE influx into wheat roots.

## Conclusions

The results obtained in this study show that two biochemical mechanisms interplay in the root uptake of PHE: (i) a fast partitioning, nonmetabolic, and 'downhill' process driven by sorption, diffusion or mass flow, which takes place immediately after the transfer of wheat roots into culture solution, and (ii) a later, slow component, which is mediated by an transporter and metabolism. In the slow process, the competition between PHE and NAP during uptake exists. This study demonstrates that a carrier-mediated, energy-consuming process is involved in PHE uptake by roots of intact wheat seedlings. This information may be beneficial to govern crop contamination by PAHs, and to yield safe produce. It is also useful in remedying soils or waters polluted by PAHs with plants.

## Methods

### Chemicals

Phenanthrene and naphthalene were purchased from Fluka Chemical Corporation with a purity >97%. Their molecular weights are 178.2 and 128.2 g mol^-1^, water solubilities at 25°C are 7.3 and 247.3 μmol L^-1 ^[26].

### Plant preparation and growth conditions

Wheat (*Triticum acstivnm L*.) seeds germinated on moist filter paper for 4 d at 25°C in the dark after surface sterilization in 10% H_2_O_2 _for 10 min and thorough rinse with Millipore (Milli-Q, Billerica, MA, USA) water. The nine uniform-sized seedlings were transplanted per 600-mL beaker wrapped with black plastic and containing 500 mL half-strength aerated Hoagland nutrient solution for 5 d and then transferred to the full-strength Hoagland solution for 5 d. The nutrient solution prepared with Millipore water and the initial pH of the solution was adjusted to 5.5. Wheat seedlings were grown in a climate chamber under controlled conditions (photoperiod 16 h light/8 h dark; light intensity 400 μmol m^-2 ^s^-1^; day/night temperature of 25/20°C; relative humidity 60%). After a 10-d growth in Hoagland nutrient solution, the wheat seedlings were immersed in Millipore water for 24 h and then employed in the subsequent phenanthrene uptake experiments.

### Time course of root uptake of phenanthrene

Nine intact 14-d-old wheat seedlings were immersed with their roots in a 600-mL beaker containing 500 mL aerated complete Hoagland nutrient solution (pH 5.5) with 5.62 μM (1.0 mg L^-1^) phenanthrene and 0.1% methanol. Seedlings were allowed to accumulate phenanthrene at 25°C for 2, 4, 8, 16 or 36 h. At each sampling point, three beakers were removed for analyzing the phenanthrene uptake by wheat roots.

### Concentration-dependent uptake of phenanthrene

As above, batches of intact wheat seedlings were transferred to 600-mL beakers containing 500 mL full-strength Hoagland nutrient solution (pH 5.5) with 0.1% methanol. The uptake of phenanthrene was detected after 4 h of uptake in the solutions with phenanthrene at concentrations of 0, 1.12, 2.25, 3.37, 4.49, 5.62 and 6.74 μM. The concentration-dependent uptake experiment was performed at 4°C and 25°C. In order to keep low temperature (4°C), ice-bath was employed [39,43]. There are triplicates in each treatment. Transpiration was measured gravimetrically using plant-free pots as controls, and the difference between 4°C and 25°C in transpiration was negligible within 4 h. Under the experiment conditions, the difference in phenanthrene apparent solubility between 4°C and 25°C was trifling.

### Phenanthrene uptake by wheat roots with or without 2,4-dinitrophenol

This experiment was conducted at 25°C. The procedures were the same as those in concentration-dependent uptake of phenanthrene. The concentration of 2,4-dinitrophenol in Hoagland nutrient solution was 1 mM [27,43].

### Uptake competition between phenanthrene and naphthalene

In uptake competition experiment, there were three treatments: a) addition of naphthalene at a concentration of 3.91 μM (0.5 mg L^-1^); b) addition of phenanthrene at 2.81 μM (0.5 mg L^-1^); and c) addition of naphthalene and phenanthrene at 3.91 and 2.81 μM. The experiment was carried out as described in concentration-dependent uptake of phenanthrene.

### Change in pH during phenanhtrene uptake

Hoagland nutrient solution and Millipore water were employed to test the change in pH during uptake of phenanthrene. Wheat roots were exposed to three phenanthrene levels, 0, 2.81 and 5.62 μM, with each treatment being replicated three times. The procedures were the same as uptake competition experiment. After 4 h of uptake, pH values of Hoagland nutrient solution and Millipore water were measured with a pH meter.

### Extraction and analysis of phenanthrene and naphthalene

After harvest, wheat roots were rinsed with methanol for about 10 seconds, and then washed with sufficient Millipore water to remove the phenanthrene and naphthalene on root surface, followed by wiping with tissue paper [46,47]. Wheat roots and shoots were weighed and ground in a glass homogenizer. Homogenized tissue samples were extracted in acetone/hexane (1:1, v/v) mixture by ultrasonication repeated three times (30 min each time). The combined solvent extracts were passed through an anhydrous Na_2_SO_4 _column with elution of the 1:1 mixture solvent of acetone and hexane. The eluents were then evaporated to dryness at 35°C in a rotary evaporator and dissolved in 12 mL hexane. Subsequently, the 12-mL solvent was cleaned in a 2-g silica gel column and eluted with 25 mL hexane/dichloromethane (1:1, v/v) solvents. The eluents were evaporated to dryness again and exchanged to 2 mL methanol. Prior to analysis of phenanthrene and naphthalene by high performance liquid chromatography (HPLC) with ultraviolet (UV) and fluorescence detection, all extracts were filtered with 0.22 μm filter units [4,48]. The mean recoveries of phenanhtrene and naphthalene acquired by spiking wheat samples with standards were 97% and 86%, respectively, for the entire procedure. None of the data reported here have been corrected for recovery. PAH contents in wheat tissues and pH values in Hoagland nutrient solution were subjected to variance analysis and statistically compared in the light of the Duncan's test at the 0.05 probability level. The inhibitions of low temperature and DNP were statistically compared according to the paired *t*-test at 95% confidence level.

The HPLC system employed consisted of an automatic injector (Waters 717), a binary high-pressure pump (Waters 1525), a UV detector (Waters 2487), and a fluorescence detector (Waters 2475). Separations were performed with a reverse phase Symmetry C_18 _(ø 4.6 × 150 mm, 5 μm particle) column. The temperature of the HPLC column was kept constant at 30°C. The used mobile phase was methanol and Millipore water (80:20, v/v), with a flow rate of 1 mL min^-1^. The injection volume was 10 μL. Phenanthrene and naphthalene were quantified at 293.5/395 (excitation/emission wavelength) and 254 nm for fluorescence detector and UV detector, respectively.

## Authors' contributions

X-HZ, L-XZ, T-HJ and G-HX designed research; X-HZ, H-LM, L-XZ and J-RL performed research; X-HZ, H-LM, L-XZ, T-HJ and G-HX analyzed data; and X-HZ, L-XZ, T-HJ and G-HX wrote the paper. All authors read and approved the final manuscript. The authors declare no conflict of interest.

## References

[B1] Boehm PD Morrison RD, Murphy BL Polycyclic aromatic hydrocarbons (PAHs) Environmental forensics: Contaminant specific guide 2006 Burlington: Academic Press 313 337

[B2] Wild SR Jones KC Polynuclear aromatic hydrocarbons in the United Kingdom environment: a preliminary source in inventory and budget Environ Pollut 1995 88 91 108 10.1016/0269-7491(95)91052-M 15091573

[B3] Edwards NT Polycyclic aromatic hydrocarbons (PAH's) in the terrestrial environment--A review J Environ Qual 1983 12 4 427 441

[B4] Kipopoulou AM Manoli E Samara C Bioconcentration of polycyclic aromatic hydrocarbons in vegetables grown in an industrial area Environ Pollut 1999 106 369 380 10.1016/S0269-7491(99)00107-4 15093033

[B5] Fismes J Perrin-Ganier C Empereur-Bissonnet P Morel JL Soil-to-root transfer and translocation of polycyclic aromatic hydrocarbons by vegetables grown on industrial contaminated soils J Environ Qual 2002 31 1649 1656 12371182 10.2134/jeq2002.1649

[B6] SamsØe-Petersen L Larsen EH Larsen PB Bruun P Uptake of trace elements and PAHs by fruit and vegetables from contaminated soils Environ Sci Technol 2002 36 3057 3063 10.1021/es015691t 12141482

[B7] Menzie CA Potocki BB Santodonato J Exposure to carcinogenic PAHs in the environment Environ Sci Technol 1992 26 7 1278 1284 10.1021/es00031a002

[B8] Phillips DH Polycyclic aromatic hydrocarbons in the Diet Mutation Research 1999 443 139 147 10415437 10.1016/s1383-5742(99)00016-2

[B9] Briggs GG Bromilow RH Evans AA Relationships between lipophilicity and root uptake and translocation of non-ionised chemicals by barley Pestic Sci 1982 13 495 504 10.1002/ps.2780130506

[B10] Edwards NT Uptake, translocation and metabolism of anthracene in bush bean Environ Toxicol Chem 1986 5 659 665

[B11] Hart JJ DiTomaso JM Linscott DL Kochian LV Characterization of the transport and cellular compartmentation of paraquat in intact maize seedlings Pestic Biochem Physiol 1992 43 212 222 10.1016/0048-3575(92)90034-W

[B12] Bakker MI Casado B Koerselman JW Tolls J Kolloffel C Polycyclic aromatic hydrocarbons in soil and plant samples from the vicinity of an oil refinery Sci Total Environ 2000 263 91 100 10.1016/S0048-9697(00)00669-0 11194166

[B13] Watts AW Ballestero TP Gardner KH Uptake of polycyclic aromatic hydrocarbons (PAHs) in salt marsh plants spartina alterniflora grown in contaminated sediments Chemosphere 2006 62 1253 1260 10.1016/j.chemosphere.2005.07.006 16213549

[B14] Lin H Tao S Zou Q Coveney RM Uptake of polycyclic aromatic hydrocarbons by maize plants Environ Pollut 2007 148 614 619 10.1016/j.envpol.2006.11.026 17254679

[B15] Marschner H Mineral nutrition of higher plants 1995 2 London: Academic Press 6 78

[B16] Ryan JA Bell RM Davidson JM O'Connor GA Plant uptake of non-ionic organic chemicals from soils Chemosphere 1988 17 12 2299 2323 10.1016/0045-6535(88)90142-7

[B17] Paterson S Mackay D Tam D Shiu WY Uptake of organic chemicals by plants: A review of processes, correlations and models Chemosphere 1990 21 3 297 331 10.1016/0045-6535(90)90002-B

[B18] Trapp S Plant uptake and transport models for neutral and ionic chemicals Environ Sci Pollut Res 2004 11 1 33 39 10.1065/espr2003.08.169 15005138

[B19] Chiou CT Sheng G Manes M A partition-limited model for the plant uptake of organic contaminants from soil and water Environ Sci Technol 2001 35 1437 1444 10.1021/es0017561 11348082

[B20] Zhu L Gao Y Predict of phenanthrene uptake by plants with a partition-limited model Environ Pollut 2004 131 505 508 10.1016/j.envpol.2004.02.003 15261414

[B21] Yang Z Zhu L Performance of the partition-limited model on predicting ryegrass uptake of polycyclic aromatic hydrocarbons Chemosphere 2007 67 402 409 10.1016/j.chemosphere.2006.09.008 17166547

[B22] Collins C Fryer M Grosso A Plant uptake of non-ionic organic chemicals Environ Sci Technol 2006 40 45 52 10.1021/es0508166 16433331

[B23] Wild E Dent J Thomas GO Jones KC Direct observation of organic contaminant uptake, storage, and metabolism within plant roots Environ Sci Technol 2005 39 3695 3702 10.1021/es048136a 15952374

[B24] Edwards NT Ross-Todd RM Garver EG Uptake and metabolism of ^14^C anthracene by soybean (*Glycine max*) Environ Exp Bot 1982 22 3 349 357 10.1016/0098-8472(82)90027-2

[B25] Schwab AP Al-Assi AA Banks MK Adsorption of naphthalene onto plant roots J Environ Qual 1998 27 220 224

[B26] Cerniglia CE Biodegradation of polycyclic aromatic hydrocarbons Biodegradation 1992 3 351 368 10.1007/BF00129093

[B27] Bowen JE Absorption of copper, zinc and manganese by sugarcane leaf tissue Plant Physiol 1969 44 225 261 10.1104/pp.44.2.255 16657055 PMC396071

[B28] McClure PR Kochian LV Spanswick RM Shaff JE Evidence for cotransport of nitrate and protons in maize roots: I. Effect of nitrate on the membrane potential Plant Physiol 1990 93 281 289 10.1104/pp.93.1.281 16667448 PMC1062500

[B29] McClure PR Kochian LV Spanswick RM Shaff JE Evidence for cotransport of nitrate and protons in maize roots: II. Measurement of NO_3_^- ^and H^+ ^fluxes with ion-selective microeletrodes Plant Physiol 1990 93 290 294 10.1104/pp.93.1.290 16667449 PMC1062501

[B30] Hess FD Duke SO Herbicide absorption and translocation and their relationship to plant tolerances and susceptibility Weed Physiology 1985 II Boca Raton: CRC Press 191 214

[B31] Su Y Zhu Y Uptake of selected PAHs from contaminated soils by rice seedlings (*Oryza sativa*) and influence of rhizosphere on PAH distribution Environ Pollut 2008 155 359 365 10.1016/j.envpol.2007.11.008 18331768

[B32] Kvesitadze E Sadunishvili T Kvesitadze G Mechanisms of organic contaminants uptake and degradation in plants World Acad Sci Eng Technol 2009 55 6 458 468

[B33] Gao Y Ling W Wong MH Plant-accelerated dissipation of phenanthrene and pyrene from water in the presence of a nonionic-surfactant Chemosphere 2006 63 1560 1567 10.1016/j.chemosphere.2005.09.058 16581106

[B34] Burton JD Balke NE Glyphosate uptake by suspension-cultured potato (*Solanum tuberosum and S. brevidens*) cells Weed Sci 1988 36 146 153

[B35] Denis M Delrot S Carrier-mediated uptake of glyphosate in broad bean (*Vicia faba*) via a phosphate transporter Physiol Plant 1993 87 569 575 10.1111/j.1399-3054.1993.tb02508.x

[B36] Tilquin M Peltier JP Marigo G Mechanisms for the coupling of iron and glyphosate uptake in Catharanthus roseus cells Pest Biochem Physiol 2000 67 145 154 10.1006/pest.2000.2487

[B37] Gao Y Zhu L Plant uptake, accumulation and translocation of phenanthrene and pyrene in soils Chemosphere 2004 55 1169 1178 10.1016/j.chemosphere.2004.01.037 15081757

[B38] Hart JJ DiTomaso JM Linscott DL Kochian LV Investigations into the cation specificity and metabolic requirements for paraquat transport in roots of intact maize seedlings Pestic Biochem Physiol 1993 45 62 71 10.1006/pest.1993.1008

[B39] DiTomaso JM Hart JJ Kochian LV Transport kinetics and metabolism of exogenously applied putrescine in roots of intact maize seedlings Plant Physiol 1992 98 611 620 10.1104/pp.98.2.611 16668685 PMC1080234

[B40] Hart JJ Norvell WA Welch RM Sullivan LA Kochian LV Characterization of zinc uptake, binding, and translocation in intact seedlings of bread and durum wheat cultivars Plant Physiol 1998 118 219 226 10.1104/pp.118.1.219 9733541 PMC34859

[B41] Schmid WE Haag HP Epstein E Absorption of zinc by excised barley roots Physiol Plant 1965 18 860 869 10.1111/j.1399-3054.1965.tb06945.x

[B42] Chaudhry FM Loneragan JF Zinc absorption by wheat seedlings and the nature of its inhibition by alkaline earth cations J Exp Bot 1972 23 552 560 10.1093/jxb/23.2.552

[B43] Liang Y Si J Römheld V Silicon uptake and transport is an active process in *Cucumis sativus* New Phytologist 2005 167 797 804 10.1111/j.1469-8137.2005.01463.x 16101916

[B44] Williams LE Lemoine R Sauer N Sugar transporters in higher plants - a diversity of roles and complex regulation Trends in Plant Sci 2000 5 7 283 290 10.1016/S1360-1385(00)01681-2 10871900

[B45] Noiraud N Maurousset L Lemoine R Transport of polyols in higher plants Plant Physiol Biochem 2001 39 717 728 10.1016/S0981-9428(01)01292-X

[B46] Schwab AP Al-Assi AA Banks MK Adsorption of naphthalene onto plant roots J Environ Qual 1998 27 220 224

[B47] Jiao X Xu F Dawson R Chen S Tao S Adsorption and absorption of polycyclic aromatic hydrocarbons to rice roots Environ Pollut 2007 148 230 235 10.1016/j.envpol.2006.10.025 17182157

[B48] Hartmann R Polycyclic aromatic hydrocarbons (PAHs) in forest soils: Critical evalution of a new analytical procedure Int J Environ Analyt Chem 1996 62 161 173 10.1080/03067319608027062

